# What’s charisma got to do with it? Three faces of charismatic leadership and corporate social responsibility engagement

**DOI:** 10.3389/fpsyg.2022.829584

**Published:** 2022-07-22

**Authors:** Jinyu Hu, Tanurima Dutta

**Affiliations:** Management Department, College of Business, University of Nevada, Reno, Reno, NV, United States

**Keywords:** corporate social responsibility, socialized charismatic leadership, personalized charismatic leadership, decision frame, CSR orientation, CSR configuration, CSR heterogeneity, upper echelons theory

## Abstract

In response to both internal and external expectations and pressures, companies increasingly consider corporate social responsibility (CSR) as an essential factor in their strategic planning, but in a very diverse manner. To help synthesize the flourishing research in CSR variation across firms, we propose a three-orientation framework to map out a wide range of CSR strategies in current literature. Furthermore, we emphasize the importance of executive leadership and suggest that differences in leader’s values are the key drivers of CSR heterogeneity. This study offers a parsimonious model that maps out three primary pathways between leadership values and CSR strategic configurations. Drawing from charismatic leadership theory, we argue that three distinct types of leader power motives define three modes of leader’s strategic decision frames, which, in turn, influence corresponding CSR orientations. Specifically, socialized charismatic leaders favor prosocial decision frame that results in integrative CSR orientation; neutralized charismatic leaders embrace instrumental decision frame leading to strategic CSR mode; and personalized charismatic leaders tend to adopt self-serving CSR strategies driven by the self-serving decision frame. This holistic view advances the knowledge about the micro-foundations of CSR drivers and the essential role of leader values.

## Introduction

Companies and their leaders increasingly acknowledge the critical role businesses play in addressing environmental and societal issues (Barney and Rangan, 2019) and allocate resources for CSR programs (Jamali and Karam, 2018). In 2019, chief executive officers (CEOs) of 181 major companies in the U.S. issued a statement reevaluating the purpose of a corporation to include a fundamental commitment to all stakeholders. These reimagined corporate objectives also highlight the inextricable tensions between firm’s pursuit of doing well and doing good by delivering values to diverse stakeholders. In seeking to balance among the complex and sometime competing expectations from various stakeholders, organizations often adopt very different CSR strategies (Godfrey et al., 2009; Bundy et al., 2018).

Such diversity in CSR engagement and its complex performance implications pose challenging questions for strategy scholars. We witness an increasing research effort in investigating the heterogeneity in company’s CSR engagement (Wang et al., 2016; Vishwanathan et al., 2020; Zhao et al., 2022). This line of inquiry tends to focus on two key questions: *how* firms are different in their CSR investments and, more recently, *why* firms adopt such diverse engagement strategies. For the questions of *how* firms differ, multiple CSR typologies (i.e., internal-external; Farooq et al., 2017; technical-institutional CSR; Mattingly and Berman, 2006) have emerged. These frameworks depict the differences in CSR postures in terms of various subgroups of stakeholders or CSR dimensions targeted by organizations’ social investment (Chang et al., 2014). In turn, these different CSR types have varying implications related to firm’s risk, social evaluation, and performance (Godfrey et al., 2009). For instance, Tang et al.’s (2015) study shows that a strategy focusing more on internal than external CSR leads to better performance than one engaging more external CSR. This is a useful lens and steps forward from using an aggregated CSR score for understanding *how* firms differ in their CSR activities. In the meantime, the typology approach also presents two limitations. One has to do with the potential typology proliferation in order to capture the full scope of combinations of stakeholder sub-groups or CSR dimensions that different firms choose to include in their CSR portfolios. In addition, CSR typology literature has yet to fully address the critical question of *why* organizations strategically prioritize some stakeholders over others and the conversations have predominantly focused on the macro and institutional drivers. As a result, such an effort to understand the heterogeneity in CSR engagement has mostly ignored the role of human decision-makers and thus almost remains “faceless.” With the voice of the key decision-makers muted, the understanding of *why* companies opt for dissimilar CSR strategies remains incomplete. Thus, an overarching configuration framework can be helpful to organize widely diverse CSR postures and shed light on underlying drivers.

To better understand CSR heterogeneity, strategy scholars argue that it is essential to examine the question of *why*, i.e., organization’s motivation underlying their CSR participation (Doh and Stumpf, 2005; Maak and Pless, 2006; Waldman, 2011; Aguinis and Glavas, 2012). A recent stream of research in strategy shifts the conversation to highlight the strategic role of CEOs and top management team (TMT) in CSR engagement. Drawing from upper echelons theory (UET; Hambrick and Mason, 1984; Hambrick, 2007), these studies portray strategic decision-makers being responsible for the diversity in CSR strategic choices (Waldman and Javidan, 2009; Wowak et al., 2016). Scholars stipulate that strategic leaders’ (CEOs and TMTs are referred as strategic leaders throughout the rest of the paper) individual attributes and psychological orientations have profound effect on organization’s strategic actions and performance. In many ways, an organization’s strategic choices are often a reflection of managerial personal values. In the case of CSR engagement, studies have shown that CEO’s personal characteristics such as political ideology (e.g., Chin et al., 2013), self-regulatory focus (Perez-Batres et al., 2012), and narcissistic personality (Petrenko et al., 2016) have a meaningful influence on CEO’s interpretation of environmental factors and choices of CSR strategies. Evidence has supported the links between certain styles of leadership and firm’s CSR engagement (e.g., authentic leadership-CSR, Kim et al., 2018; ethical leadership-CSR, Saha et al., 2020). This line of inquiry provides critical insight into the role of decision-maker’s personal values in firm’s CSR diversity. However, current leader-CSR research has a similar limitation as the CSR typology literature. These studies primarily focus on piecemealed CEO-CSR links (i.e., one attribute-one type of CSR, one leader style-one type of CSR). While acknowledging the research effort in exploring a wide range of leader style-CSR links, we also see a need for an overarching framework to address the more encompassing research question: *What are the core leadership principles underlying various leadership styles that drive different CSR choices*?

Taken together, we see rich but fragmented research streams in both the *how*-literature (CSR typology with stakeholder lens) and the *why*-literature (CSR drivers with UET lens). Time is ripe for developing a more holistic understanding of *why and how* companies manage CSR differently. Our study addresses the research gap discussed above by proposing an overarching framework to coherently synthesize the leadership-CSR literature. The central premise is that firm’s CSR activities are executive leader’s strategic choices influenced by leader’s personal values. Leaders have different value systems, particularly those associated with power and sense of responsibilities for others. As a result, we see different modes of CSR engagement.

There are two main objectives here. One is to develop an *encompassing framework* to synthesize the wide range of leader behaviors and CSR strategies in the literature. An impressive number of studies have provided enormous insights into the Leader-CSR phenomena (Zhao et al., 2022). A number of systematic review pieces have done the field a great service by summarizing the leadership-CSR literature with grand details and breadth (e.g., Pless et al., 2012; Miska and Mendenhall, 2018; Ashrafi et al., 2020; Saha et al., 2020; Zhao et al., 2022). This is also where our paper departs from these studies. Thus, our second objective is to build an overarching conceptual model to integrate the extant literature on leader-CSR. The unique contribution of this study is the *parsimonious synthesizing theme:* we address the question of how leadership impacts CSR strategies by identifying the CSR-related value principles underpinning various leader styles (opp. Specific leader style in relation to particular CSR tactic). Similarly, we identify three high-order families of CSR orientations to represent the principal characteristics of diverse CSR portfolios. Furthermore, we highlight the CSR decision frame as an underlying mechanism and develop the pathway model linking leadership to CSR. Specifically, leader’s power motives are translated into his or her CSR decision frame, which in turn defines leader’s interpretations of the environment and assessment of various stakeholders (Mitchell et al., 1997) and ultimately firm’s CSR preferences.

**Figure 1:**
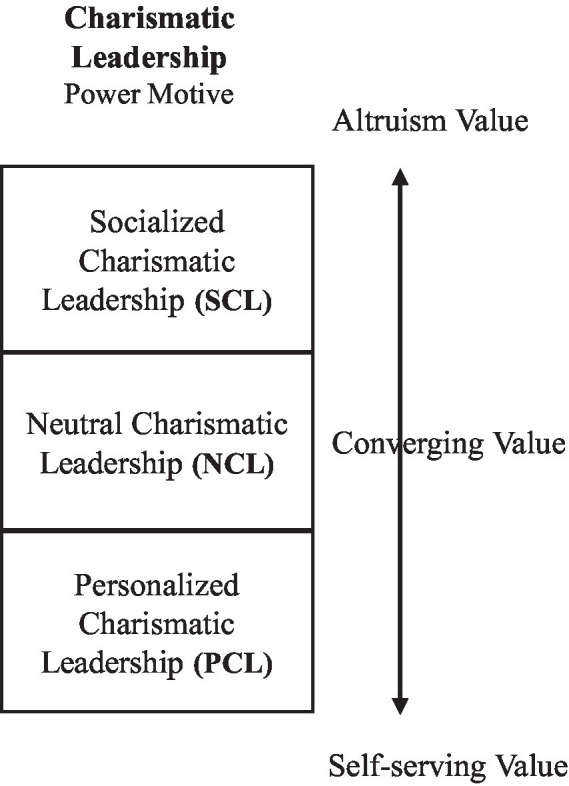
Leader value continuum.

**Figure 2:**
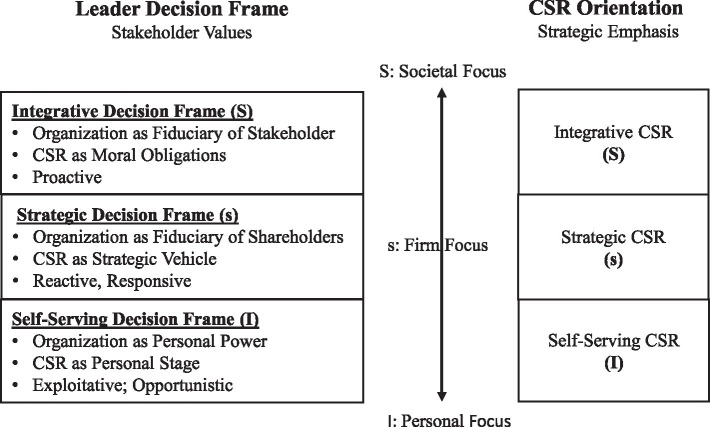
Stakeholder value continuum.

To achieve such encompassing yet parsimonious dual objective, we adopt a spectrum approach to conceptualize leadership values, CSR decision frames, and CSR orientations as three continuums, respectively, (as shown in Figures 1, 2). We then define three focal points along each spectrum to articulate the key distinctions among core principles. Along the leader-value spectrum, there are three types of power motives (three “faces”), altruism value, converging value, and self-serving value. Similarly, along the spectrum for leader decision frame and CSR orientation, there are three types of foci including societal focus, firm focus, and personal focus. These focal points provide a parsimonious structure along the encompassing spectrum. In essence, diverse leadership styles can be synthesized into three CSR-related value systems, while diverse CSR strategies are summarized into three primary orientations.

**Figure 3:**
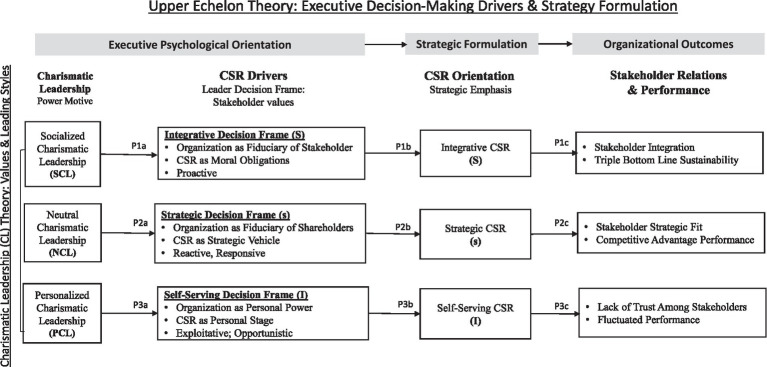
Three-pathway model of corporate social responsibility (CSR).

To build a coherent pathway model, we draw from the following theoretical lenses: charismatic leadership theory (House, 1977; House and Howell, 1992; Shamir and Howell, 2018), which defines leader values, and CSR strategic modes (e.g., Aguilera et al., 2007; Pless et al., 2012; Carter and Greer, 2013; Miska et al., 2014; Waldman, 2014; Maak et al., 2016; Gond et al., 2017), a foundation for the concept of CSR orientation. Charismatic leadership theory is an influential value-based leadership framework popular in the micro-discipline (organizational behavior). There are two compelling reasons for the adoption. First, this is one of the few leadership theories that emphasizes the role of values (particularly related to power motives and responsibility for others; Pless and Maak, 2011; Miska et al., 2014). This aligns with the essence of CSR strategic choice, which is about how leaders perceive their responsibility, through the lens of personal values, toward various stakeholders (Mitchell et al., 1997). In addition, charismatic leadership is also the only leadership theory that articulates a full range of values covering both the self-serving and prosocial ends of the spectrum. This multi-dimensional feature enables us to coherently synthesize diverse leader value systems, particularly those related to CSR beliefs on one continuum (Howell and Shamir, 2005; Watts et al., 2018). Thus, instead of considering charismatic leadership theory as a framework of leader styles (e.g., authentic leadership, servant leadership), we adopt it as a model of leader value systems, which provides a parsimonious structure to compare and contrast the good, the bad, and the ugly of diverse leaders’ power motives (Devinney, 2009). We suggest that strategic leaders with different power motives are likely to adopt different CSR decision frames (Hu et al., 2022). As a result, firm’s CSR strategies fall under one of the three orientations: integrative CSR mode (S), strategic CSR mode (s), and self-serving CSR mode (I; Figure 3).

In summary, with this three-pathway model, we contribute to the research on the important role of executive leaders in CSR heterogeneity in the following ways.

First, this study provides a parsimonious framework to synthesize the rich literature on leader-CSR links. Particularly, we identify three types of leader power motives and three CSR orientations representing the primary attributes underlying a wide range of leader styles and CSR modes, respectively. Furthermore, three key focal points are placed along each spectrum to mark the prominent distinctions across different types of principles (“three faces”: Figures 1, 2). These focal points also illustrate the “gray-zones” in-between focal points, which differ in terms of degrees. Thus, with a spectrum approach, we are not scarifying the complexity and nuances of the wide range of leader styles and CSR strategies.

Secondly, we conceptualize the CSR decision frame as the cognitive lens strategic leaders are likely to adopt to guide their strategic decision-making process (Hu et al., 2022). This idea provides an important mechanism underlying leader’s CSR preferences and helps us build a pathway model. Specifically, we identify three fundamental questions associated with CSR (i.e., purpose of firm, utility of CSR, and leader responsibility). Different leader power motive drives different answers to these three principal questions, which, in turn, defines three focal points along the continuum for CSR decision frame orientations. CSR decision frames define shareholder values and salience for leaders and guide leader’s attention in strategy formulation and priorities in resource allocation. We believe that this CSR decision framing idea (Hu et al., 2022) contributes to the call for a better understanding of CEO’s CSR decision deliberations (Zhao et al., 2022) and the underlying mechanisms of CSR (Aguinis and Glavas, 2012). It explicitly addresses the question of *how* executives make CSR decisions.

Thirdly, in responding to the voice of prominent scholars (Aguinis and Glavas, 2012), this study bridges the micro- and macro-focused development in CSR research. We place a human face to business’ CSR strategic choices by illuminating the role of their power motives and decision frames. This micro-conceptualization fits well with the overarching assumptions of the UET (Hambrick and Mason, 1984; Hambrick, 2007), which emphasizes the critical role of executive psychological orientation. Leader’s power motive and decision frame concepts expand current proxies for executive’s psychological orientation in UET research on CSR.

Lastly, as an alternative to existing stakeholder-based CSR typologies (Pless et al., 2012; Maak et al., 2016), we propose a consolidatory S-s-I framework that depicts the heterogeneity in CSR strategic configurations. Specifically, three types of power motives (socialized, neutral, and personalized power motives) predict three corresponding decision frames (integrative/S, instrumental/s, and self-serving/I decision frames), and consequently, three CSR orientations (integrative/S, Strategic/s, and Self-serving/I CSR configurations). These three sets of typologies are a complementary expansion of current CSR typology literature with an emphasis on the dynamic and multifaceted nature of stakeholder relationship management.

In the following sections, we will first outline the theoretical assumptions of charismatic leadership theory (Table 1), followed by articulating three types of power motives and corresponding strategic decision frames. We then build the three pathways linking the S-s-I value profiles with the corresponding S-s-I CSR strategic orientations (Figure 1).

**Table 1:** Distinction between socialized and personalized charismatic leadership.

Dimensions	Socialized charismatic leadership (SCL)	Personalized charismatic leadership (PCL)
Values	Ethicality, integrity; moral altruism; prosocial values.	Unethicality; personal dominance; manipulation; antisocial values.
Vision	Serves collective interests.	Serves self-interests.
	Focus on the greater good.	Focus on personal gain and impact.
	Inspirational.	Self-serving.
Power motive	Self-control, constraint; humility	Lack of self-control; narcissism.
Activity inhibition	Egalitarian.	Authoritative.
Leadership styles	Responsible leadership.	Self-serving; exploitative leadership.

## Theoretical development

### Charismatic leadership and its three faces

Charismatic leaders are often considered “visionary” and “exceptional” and are capable of influencing others by “engendering excitement towards a common cause” (Wowak et al., 2016, p. 586). Such extraordinary influence is derived from their personal charisma, defined as a value-laded individual characteristic and a personal quality, which enables them to have a profound social influence on others (House, 1977; Antonakis et al., 2016). The evidenced effectiveness of this leadership style can be credited to individual’s ability to promote their ideology with often unconventional means to achieve changes (Banks et al., 2017). According to the charisma literature, a charismatic leader’s strategic decisions are shaped by the underlying individual values trademarked as boldness, resistance to the status quo, and high self-confidence (Waldman and Javidan, 2009; Wowak et al., 2016). Numerous studies have found that charismatic CEOs are more likely to engage in strategic initiatives associated with novelty, change, and CSR (Waldman et al., 2001; Luque et al., 2008; Wowak et al., 2016; Banks et al., 2017).

A principal feature of the charismatic leadership theory is that charisma is considered a double-edged sword (Waldman and Javidan, 2009; Vergauwe et al., 2018). Howell and Avolio (1992) pioneered the concepts of personalized and socialized charismatic leadership (SCL) to differentiate between the good or moral side and the evil or immoral side of value-laded power motives. Socialized charismatic leaders are motivated by a “socialized power orientation” and inspire people to strive for prosocial goals by sacrificing personal interest (O’Connor et al., 1995; Walter and Bruch, 2008; Varella et al., 2012), while personalized charismatic leaders are driven by a “personalized power orientation” that focuses on personal ambitions at the expense of collective interests (Howell and Shamir, 2005; Watts et al., 2018). Depending on where it falls on the value spectrum, charismatic leaders can mobilize people to pursuing either socially destructive or constructive objectives (Waldman and Javidan, 2009; Watts et al., 2018).

Along the charismatic leadership value continuum (Figure 1), we identify three focal points, SCL, NCL, and PCL, to articulate the key differences in various leader power motive orientations.

#### Socialized charismatic leadership

The essence of SCL lies in their high moral standards and integrity (Avolio et al., 2004; Waldman, 2014). Our characterization of socialized charismatic leader values is largely drawn from the accumulated work on SCL (e.g., House and Howell, 1992; Waldman and Yammarino, 1999; Waldman and Javidan, 2009; Antonakis et al., 2016; Wowak et al., 2016; Shamir and Howell, 2018). According to this stream of research, socialized charismatic leaders are guided by ethicality and morally altruistic principles. Prior research has drawn an association between moral altruism and prosocial values (Waldman and Javidan, 2009; Varella et al., 2012). In fact, such association is manifest in the overall outlook of socialized charismatic leaders which is shaped by their *prosocial values*.

Humility appears to be strongly correlated with socialized leader behavior (Maccoby, 2004; Humphreys et al., 2010; Nielsen et al., 2010; Ou et al., 2018). Comte-Spoonville (2001) suggested that humility should be thought of as the science of the self because it signifies the propensity to develop a thorough understanding of the self. Discovery of individual strengths and weaknesses accompanies the process of gaining such understanding guided by the search for the ultimate truth.

Built upon current literature on SCL, we summarize the major distinction between personalized and socialized leaders in Table 1 and emphasize how they create and articulate their *vision* differently. Unlike personalized charismatic leaders who are guided by their self-interest, socialized charismatic leaders serve collective interests and are genuinely concerned about societal good (Wowak et al., 2016). As a result, they express an inspirational vision that is aligned with the interests and desires of the followers (Howell and Avolio, 1992; Shamir and Howell, 2018).

Additionally, the differences between personalized and socialized charismatic leaders are also observable in light of the nature of their *power motives*. Socialized charismatic leaders have a socialized power motive that is concomitantly shaped by the level of their need for power and activity inhibition. According to the Leader Motive Profile theory (LMP; McClelland, 1985, 1992; Miner, 1993; Winter, 2001; Fodor, 2010), “effective leaders will both enjoy the process of exerting a social influence (need for power) and avoid using power in an exploitive manner through coercion or manipulation (*activity inhibition*)” (House and Howell, 1992, p. 90). The major implication of these two forces is the manner in which the leader satisfies the need for power (Waldman et al., 2006; Weinberger et al., 2010). Whether such need is satisfied in a prosocial way or antisocial way, depends on the leader’s (un)willingness to take responsibility for his actions and for the consequences of such actions on others (Winter, 2001; Waldman and Javidan, 2009; Watts et al., 2018). To that end, we conclude that socialized charismatic leaders have a high need for power and a high level of activity inhibition. In other words, a high need for power combined with a high level of activity inhibition drives socialized charismatic leaders to seek power for serving the greater good for society. With a self-controlled power motive, these leaders apply restraint in the use of their power and direct it toward social responsibility instead of personal gain (House and Howell, 1992; Vergauwe et al., 2018).

Furthermore, contrary to personalized charismatic leaders, socialized charismatic leaders are humble because they do not feel the need to project a grandiose image to their followers. Current research also shows that humble leaders are willing to assimilate new ideas and encourage others to express their opinions (Tangney, 2000; Ou et al., 2018). This is another way of saying that humility allows socialized charismatic leaders to transcend the self and consider the world and the larger reality (Nielsen et al., 2010).

In a nutshell, socialized charismatic leaders espouse egalitarianism and genuine concern for a wide array of stakeholders. Driven by a compelling vision which is responsive to the stakeholder’s needs, they endeavor to cognitively connect and engender an inspirational impact (Waldman and Yammarino, 1999; Wowak et al., 2022). Additionally, by using power in socially constructive ways, they contribute to the welfare of the organization and beyond (Waldman et al., 2006; Waldman, 2014).

#### Personalized charismatic leadership

As mentioned earlier, there is a potential immoral and wicked aspect of charismatic leadership which is represented by the personalized charismatic leadership (SCL) values. Unlike socialized charismatic leaders, personalized charismatic leaders’ *values* are based on personal dominance and authoritative behavior (Winter, 2001; Watts et al., 2018). They are self-aggrandizing and promote their personal agenda by engaging in one-way communication with their followers. They pursue goals in congruence with their self-interest and manipulate the needs of the followers and the organization to fulfill those interests (Braun, 2017).

Researchers have also studied SCL in the context of the *vision* of such leaders (House et al., 1991; Waldman and Javidan, 2009; Boone et al., 2020). The fundamental characteristic of their vision is the development of such vision from their personal self. As a result, there is no alignment of their personal vision with the needs and aspirations of employees and stakeholders, and the vision stresses on the leaders’ self-interest, personal gain, and impact.

Research on charisma has shown that personalized *power motive*, which is the essence of personalized charismatic leaders, is shaped by their high need for power and a low level of activity inhibition (House and Howell, 1992; Waldman and Javidan, 2009). In this case, this type of leaders has a voracious hunger for power and influence. Equipped with a low activity inhibition which is indicative of low self-control, they direct power toward their personal benefit only and show a lack of genuine concern for the greater good. Therefore, it is unlikely that personalized charismatic leaders will appeal to prosocial values which form the crux of most CSR initiatives (Petrenko et al., 2016; Braun, 2017). Instead, they are guided by antisocial values which influence them to act in a self-serving manner (Petrenko et al., 2016). Thus, even if they express any interest in the pursuit of seemingly prosocial activities, their commitment toward such activities will be “marginal and purely calculative” (Waldman et al., 2006, p. 1719).

The literature on personalized charisma reveals narcissism as a core personality aspect of such leaders (Popper, 2002; Humphreys et al., 2010). Narcissism is associated with individual self-confidence, aggression, and managerial and autocratic tendencies (Chatterjee and Hambrick, 2011; O’Reilly et al., 2014). The fact that personalized charismatic leaders have disregard for their followers’ needs signifies a lack of empathy which is also associated with narcissism (Tang et al., 2018). Moreover, personalized charismatic leaders *exhibit* a morally righteous “image” to enhance their influence and elevate their social status (Chen et al., 2021). Such behavioral characteristics of exhibitionism and social assertiveness further corroborate their narcissistic tendencies.

In summary, personalized charismatic leaders exhibit self-serving and *autocratic style* in their approach to leading. Pursuit of wealth, power, and winning at all costs signify their heightened insensitivity to employees’ needs and aspirations (Van Scotter, 2020). Given that personalized leaders induce employees and other organizational members to comply with their personal wishes, it is unlikely that they empower employees or encourage them to think in novel ways.

#### Neutral charismatic leadership

Recent writing on charismatic leadership has extended beyond the traditional dichotomous conceptualization of personalized versus socialized power motive (Howell and Avolio, 1992; Waldman and Javidan, 2009) and favors a continuous spectrum view (e.g., Miska and Mendenhall, 2018; Watts et al., 2018). It suggests that the distinctions between personalized and SCL are unlikely a simplistic clear-cut. Rather, leaders reside along a continuum and are affected by these power motives to varying degrees (Waldman and Javidan, 2009; Watts et al., 2018). Leaders may demonstrate PCL behaviors sometimes while SCL other times. Incorporating this perspective, we introduce a “neutral” position on the personalized-socialized charisma continuum to capture the middle of the road power-motive orientations and associated behaviors.

The mid-point between socialized and personalized values represents a converging or blend of the two more extreme cases. This type of power motive is less altruistic but also less self-interest driven as well relative to SCL and PCL, respectively. Such a value system can be captured well by the traditional strategic management concept where leaders consider themselves as the agent of the principles (business owners and shareholders). They view themselves to be powerful owning to their job title and their control over resources. But, they are also aware of the boundaries of such power, which is to function within the laws and comply with regulations and social norms to serve organizational goals. NCL is driven by optimizing individual goals, which are aligned with the bottom line and success of the organization by design (e.g., corporate governance and reward structures). In this sense, NCL has limited self-interest due to the fact that, as an agent of shareholders, their success is judged and fulfilled by how well they can deliver the economic performance for the firm (Friedman, 2007). NCL sees their job beginning and ending with the organization (Waldman and Galvin, 2008) and their fundamental responsibilities as balancing tradeoffs and reconciling competing demands on organization resources (Waldman et al., 2020).

#### SCL, NCL, and PCL

In summary, CSR represents the pinnacle of the increasing complexity confronted by organizations, where leaders seek to balance between economic goals and environmental and social interests. Organizations rely on how well strategic leaders are equipped to manage these often-ill-defined situations, uncertainties, and potential chaos (Uhl-Bien et al., 2007; Samimi et al., 2022; Zhao et al., 2022). Thus, understanding leaders and their driving principles is essential. We argue that charismatic leadership theory (House, 1977; House and Howell, 1992) is well suited to investigate leader motivations. Studies have consistently shown that charismatic leaders influence firm strategy to the extent that the leaders’ values and motives determine the leadership styles (Waldman et al., 2006; Waldman and Javidan, 2009; Wowak et al., 2022). Thus, the three-faced (PCL/NCL/SCL) charismatic leadership framework provides a parsimonious organizing framework to capture a wide range of leadership behaviors along the social-self-serving value spectrum.

Furthermore, we argue that personal values function through guiding information processing and decision-making (Kurucz et al., 2008; Fabrizi et al., 2014). In the following section, we introduce the concept of leader CSR decision frame as the key mechanisms underlying the link between leader values and CSR choices.

### Leader’s decision frame

Throughout this study, we define CSR as the “actions on the part of the firm that appear to advance or acquiesce in the promotion of some social good, beyond the immediate interests of the firm and its shareholders and beyond that which is required by law” (Davis, 1973, p. 312; Waldman et al., 2006, p. 1703). We will focus on CSR as those voluntary activities that are not legally required. These discretionary CSR choices require strategic leaders to go above and beyond legal compliance. Being confronted with accountability toward both internal and external stakeholders, executives’ choices made to deal with intricacy, complexity, and uncertainty are more likely the expression of their personal characters and conviction. Further, we propose that these personal values will likely be translated into leaders’ decision frames, a cognitive lens or mental model that, in turn, guides how leaders interpret information and assign primacy scores to various issues and interest groups (Hambrick and Wowak, 2021; Hu et al., 2022; Samimi et al., 2022).

Decision frame has its root in Tversky and Kahneman’s (1981) work and refers to the “mental states primed by situational factors that influence how people evaluate and make complex decisions” (Watts et al., 2018, p. 277). In the context of CSR, the decision frame captures leaders’ mental model specifically related to CSR strategic decisions (Windsor, 2012; Hu et al., 2022). CSR decision frame is defined by the answers to these three fundamental questions relevant to CSR: the purpose of an organization, utility of CSR, and the responsibility and accountability of strategic leaders. Different answers lead to different CSR decision frames (Hu et al., 2022). In line with the spectrum approach, these diverse leader CSR decision frames are thought to reside along a continuum. We identify three focal points on the spectrum to represent three main types of CSR decision frames (S-s-I): *integrative decision frame (S), instrumental decision frame (s), and self-serving decision frame* (I). This decision frame typology captures leaders’ varied understandings of the principal issues associated with CSR, which will influence how strategic leaders see and interpret the challenges and demands of CSR and the salience and priority of shareholders (i.e., shareholder values; Voegtlin et al., 2012; Hu et al., 2022).

In the meantime, we stipulate that leader’s principal belief contributes to the development of decision frame, which, in turn, influences how leaders interpret key information related to CSR issues (Boone et al., 2020; Zhao et al., 2022). In other words, CSR decision frames are expression of leaders’ personal values, beliefs, and attitudes (e.g., SCL, NCL, and PCL). Ultimately, this CSR decision frame serves as the mechanisms underpinning the pathway from leader values to company’s CSR strategy.

In the following sections, we elaborate on the concepts and effects of each of the three CSR strategic decision frames; and present three unique pathways to link the three faces of charismatic strategic leaders with the three shades of CSR orientations (Figure 1).

## Three pathways of CSR

### Pathway 1: SCL, integrative decision frame, and integrative CSR (“S”)

#### SCL: Socialized power motives and integrative decision frame

As we have previously noted, SCL is a combination of a high need for power and extraordinary ability to exercise their influence and mobilize people for socially constructive causes (House and Howell, 1992; Boone et al., 2020). Driven by socialized power motives, SCL will be more likely to adopt an integrative strategic decision frame, which addresses the three fundamental questions about the responsibilities of the organization, value about CSR, and responsibility of corporate leaders as follows. First, SCL sees organizations as corporate citizens and the fiduciary of the people, plants, and communities (Watts et al., 2018). An integrative view of the relationship between a business and its stakeholders is the defining aspect of an integrative decision frame (Aguilera et al., 2007; Marcy, 2020). Thus, SCL understands that organizational objectives go beyond economic and legal concerns and are not only about profit-maximization. Rather, business is responsible for and should be held accountable to all stakeholders; and create value for the broader society (Dmytriyev et al., 2021). In fact, SCL sees profits as a result from doing business in a purposeful and responsible way (Pless et al., 2012; Maak et al., 2016). Secondly, SCL considers organizations as social actor that bears a moral obligation of doing business responsibly including playing a critical role in solving environmental and social problems (CSR; King et al., 2010). Lastly, leaders have the ultimate moral duty to proactively formulate and execute multi-dimensional CSR strategies (Pless et al., 2012; Saha et al., 2020) and bring business interest to align with those of society (Preston, 1975; Marcy, 2020).

#### SCL: Integrative decision frame and integrative CSR

As a direct result of the socialized strategic decision frame, SCLs are multifaceted thinkers and highly sensitive to social goals. They can recognize the needs and diverse demands from complex and interconnected business environment (Pless et al., 2012; Maak et al., 2016). They appeal to prosocial and moral values that make them more likely to consider multiple stakeholders and serve collective interests when making strategic decisions (Luque et al., 2008; King et al., 2010). They will embrace a broad approach to CSR, an integrative CSR orientation (S). This type of cause-serving CSR represents “a genuine manifestation of the firm’s underlying intentions, vision and character” (Donia et al., 2017). These CSR activities are executed with sufficient resource and expertise commitment and focus on realizing true social benefits (Christensen et al., 2014). As a result, this CSR type resonates organization’s responsibility, social justice, and compassion (Devinney, 2009; Chaudhary, 2021). SCL adopts an integrative CSR mode as an ethical conviction and is likely to be construed by stakeholders as a giver working toward making a genuine contribution to the society (Donia et al., 2019; Saha et al., 2020).

#### SCL: Integrative CSR, trust-based stakeholder relationship, and triple bottom line performance

An integrative CSR portfolio, in turn, generates multi-dimensional outcomes including stakeholder relationship built on trust and triple bottom line sustainable performance (Elkington, 1997; Devinney, 2009; Christensen et al., 2014). SCL advocates prosocial values and connects with the larger audience (Antonakis et al., 2016) and is also likely to be transparent with not only internal employees but also stakeholders. Organizations’ commitment to a common cause earns public trust and helps build sustainable relationships with stakeholders. SCL’s unconventional perspective and boldness help shape innovative culture and deploy resources to achieve synergies with multiple stakeholders (Pless et al., 2012; Wowak et al., 2022). Thus, an organization can achieve business integration by building flexible business models.

In summary, socialized charismatic leaders are moral-value driven and have a strong sense of accountability toward broader constituents and stakeholders, who commit to deliver values to diverse interest groups. Thus, our first pathway stipulates that,

Pathway 1: the “S” path

(1a). SCLs are more likely to adopt integrative decision frame (S).

(1b). Leaders with an integrative decision frame (S) are more likely to engage in an integrative CSR strategy (S).

(1c). An integrative CSR (S) will generate stakeholder integration and achieve triple bottom line sustainable performance.

### Pathway 2: NCL, instrumental decision frame, and strategic CSR (“s”)

SCL and PCL are the two ends of a continuum, in our view. SCL is the representation of the ideal and altruistic end, while PCL indicates the end that is highly driven by self-interest without concerns for others. We suspect many of the strategic leaders reside along the section that falls in-between these two ends, as neutral charismatic leadership (NCL) with converging power motives.

#### NCL: Neutral power motives and instrumental decision frame

At a converging point between the prosocial and self-serving values, NCL serves as an agent of the owners/shareholders and controllers of the organization resources, thus derives power from its legitimate role and authority within the organization. Comparing with SCL, NCL tends to have a narrower lens when it comes to social betterment and considers it to be at the service of the organizational goals (Miska et al., 2014). NCL likely demonstrates a transactional and calculative cognitive style primed by cost–benefit analysis (Waldman and Galvin, 2008; Pless et al., 2012). NCL sees their personal objectives to be a perfect alignment with company effectiveness. Doing the right thing for NCL is defined by doing their job to create values for shareholders within the boundaries of laws and industry norms (Carter and Greer, 2013). Comparing with PCL who strives for *personal* gain at the expense of others NCL is other-regarding and places the highest concerns on the organization they lead (Miska et al., 2014; Waldman, 2014). Thus, we label it as an *instrumental decision fram*e with a small “s,” a mental model that emphasizes strategic focus for the company and narrower scope for social welfare.

Applying this perspective, NCL will address the three fundamental questions as follows. First, organizations serve the purpose of maximizing shareholder’s interest by delivering superb financial performance. A sustainable mission for a business is to generate long-term economic success while serving other stakeholders if and only if that is beneficial for the bottom line (Pless et al., 2012). Second, although acknowledging businesses need to respond to the expectations of multiple stakeholders, NCL considers such diverse demands as financial burdens with competing interests for firm’s bottom line, all of which need to be balanced and efficiently managed. CSR initiatives are thought to be strategic in the sense that they have the potential for helping firm manage risk, legitimacy, and reputation. In simple words, only strategic stakeholders matter for the firm. Lastly, NCL places the obligation of executives as “limited to deploying resources as effectively as possible, based on instrumental thinking, in order to maximize the wealth of the firm” (Waldman and Siegel, 2008, p. 126). Ultimately, NCL is likely to formulate CSR as a reaction to external pressures and demands arising from institutional norms.

#### NCL: Instrumental decision frame and strategic CSR

Such instrumental decision frame will guide NCL *to embrace a strategic CSR mode*. Like SCL, NCL acknowledges the needs to address diverse expectations of multiple non-financial stakeholders. However, an *instrumental decision frame* places the constraints of firm resources at the front and center and considers CSR initiative as a cost toward the bottom line. Thus, not all stakeholders have equal importance in consideration. Rather, the preference for any particular interest group as a candidate for CSR investment will be determined by their value in serving firm’s self-interests such as legitimacy, image, and economic bottom line (Waldman and Siegel, 2008). In essence, each stakeholder is assessed based on their value for generating a competitive advantage for the company.

#### NCL: Strategic CSR, stakeholders with complementary fit, and bottom line performance

As expected, *strategic CSR (s)* is economically focused and driven by organization regarding transactional motives. Put it another way, NCL invests in strategic CSR for a direct and fast return, which can be in the form of media coverage, good will, increasing demands from customer (Elfenbein and McManus, 2010), loyalty from internal employees (Flammer and Luo, 2017), or favorable assessments from investors (Cheng et al., 2014). Strategic CSR emphasizes the profit-maximizing motives of the firm (Baron, 2001; Dmytriyev et al., 2021). CSR activities are often conducted in the form of externally visible initiatives such as philanthropic donations, which benefit the firms’ strategic competitiveness by building a positive image among current or potential stakeholders and make a firm an attractive business partner (Godfrey, 2005; Vishwanathan et al., 2020). Thus, firms are reaping strategic benefits by attracting a bigger pool of partners for future business operations. In addition, strategic CSR initiatives tend to focus on existing stakeholders that are a complementary fit strategically (McWilliams and Siegel, 2001). In turn, these stakeholders reciprocate with cooperative relationships that ultimately lead to strategic competitiveness. Despite the multifaceted nature of strategic CSR that addresses demands from various stakeholders, the principle is likely to be driven by the business case of the CSR initiatives (McWilliams et al., 2006).

In summary, NCL promotes the idea of doing well by doing good. CSR serves as the means to the end of profit maximization by achieving strategic alliance with extended stakeholders than shareholders alone, all but to gain a competitive advantage for the firm (Porter and Kramer, 2006). Thus, the second pathway of CSR shows that,

Pathway 2: the “s” path

(1a). NCL is more likely to adopt an instrumental decision frame (s).

(1b). Leaders with instrumental decision frame (s) are more likely to engage in strategic CSR (s).

(1c). A strategic CSR (s) will generate complementary strategic fit among selected stakeholders and achieve a competitive advantage for the firm.

### Pathway 3: PCL, self-serving decision frame, and self-serving CSR (“I”)

Residing on the opposite end from SCL, PCL is a form of leadership that lacks concerns for the well-being and needs of others while being controlled by their inflated self-views. They often thrive by appealing to the attention and admiration of others (Chatterjee and Hambrick, 2011; Petrenko et al., 2016).

#### PCL: Personalized power motives and self-serving decision frame

The *personalized power motives* are associated with a *self-serving decision frame* that addresses the three fundamental questions in the following way. First, like NCL, PCL will likely subscribe to a result-centric view of the firm. However, the key difference for PCL is the performance of the organization along with everything else is in service of their personal goals (not the organization). This leadership style emphasizes personal dominance, status, and prestige rather than serving collective interests (Sosik, 2005). This further strengthens our argument that these leaders would not realize the complex interdependence among the firm’s various stakeholders and would thus have a narrow view of the instrumental value of CSR and stakeholders (Devinney, 2009). Secondly, PCL tends to rely on external moral standards that fluctuate for the satisfaction of self-interests (Petrenko et al., 2016; Cragun et al., 2020). Thus, CSR is considered an effective strategic tactic for exhibiting him or herself in a favorable light and thus protecting his/her winning, wealth, and power (Chen et al., 2009; Chatterjee and Hambrick, 2011). These CSR practices reap potential strategic benefits in the form of greater attention and acclaim for themselves from the media and community. In other words, CSR is a means to the end for serving PCL’s personal aspirations and gains. Lastly, PCL’s primary focus is to serve self-interests, manipulate others for their personal gain, and win at all costs (Braun, 2017). They tend to have a low activity inhibition, which means that they rarely exercise self-control and moral constraints. They tend to abuse power vested in them for the purpose of pursuing self-interests, and at the expense of others.

#### PCL: Self-serving decision frame and self-serving CSR

We propose that PCL would embrace *self-serving mode of CSR*. Specifically, PCLs would have the propensity to engage in reputation-enhancing CSR initiatives like philanthropic donations to garner praise and attention mostly for the leaders. This type of CSR is often designed to ingratiate and appease powerful stakeholders and garnish media attention for the leaders (Donia et al., 2017). Owing to their narcissistic tendencies, PCL would constantly seek to exhibit a righteous image by engaging in visible social initiatives which resonate a moral high ground (Petrenko et al., 2016). Such initiatives would provide opportunities for personalized leaders to build a grandiose image and enhance admiration, self-esteem, and legitimacy (Al-Shammari et al., 2021). It is likely that PCL would not favor CSR initiatives which are internally focused with no immediate apparent benefit to their egos.

#### PCL: Self-serving CSR, stakeholder skepticism, and fluctuated performance

A leader who engages in such symbolic and self-serving CSR merely acts as a “taker” and attempts to protect the material resources without genuinely addressing any societal concerns (Donia et al., 2017; Fox et al., 2020). This is a potential dark side to CSR in that these initiatives do not always fulfill a genuine social need (Price and Sun, 2017; Waldman et al., 2020). Due to the opportunistic and exploitative nature, the self-serving CSR initiatives might be short-term and disconnected with other programs, which can also cause fluctuation in firm performance (Braun, 2017). In fact, self-serving CSRs can harm internal and external stakeholders’ interests in the long-run and damage the trust among stakeholders. To summarize, we propose the pathway 3 for CSR as follows,

Pathway 3: the “I” Path

(3a). PCLs are more likely to adopt a self-serving decision frame (I).

(3b). Leaders with a self-serving decision frame (I) are more likely to engage in a self-serving CSR strategic mode (I).

(3c). A self-serving CSR mode (I) will lead to a stakeholder relationship lack of trust and fluctuated financial performance.

## Discussion and limitations

We set off to address the research questions of *how* and *why* related to the heterogeneity in CSR strategies. To this end, we have attempted to explain the impact of three faces of charismatic leadership styles on three orientations of CSR decision frames and three resulting CSR strategic modes. We suggest a spectrum approach and consider differences across various types to be more of degrees than a clear-but or black-and-white. Each type of key concept (charismatic leadership, CSR decision frames, and CSR strategic orientations) is conceptualized as a focal point on a continuum. The three pathways are suggested to be the representation of the predominant tendency and most likely alignment between values, decision frames, and strategic choices. Thus, we do not claim that cross-pathway alignment will not occur. Rather, in most cases, the more a leader demonstrates the characteristics of a particular type (fall on the focal point), the more likely he or she will adopt the corresponding CSR decision frame and make corresponding CSR choices.

Research examining the micro-foundations of CSR, especially the interaction between leadership styles and CSR is still nascent (Rupp and Mallory, 2015; Farooq et al., 2017; Zhao et al., 2022). Our paper makes several theoretical contributions. First, we expand research on charismatic leadership by highlighting the three faces of charisma that have not attracted considerable scholarly attention. We provide a more nuanced understanding of the differences between the understudied personalized and SCL styles by explicating their behavior, values, and motives (Waldman and Javidan, 2009; Antonakis et al., 2016). In addition, we offer explanations for the underlying mechanisms which justify why the three types of charismatic leaders differ in their CSR engagement. The second contribution lies in our attempt to research multidimensional CSR. We respond to the calls for disaggregating CSR (Wang and Choi, 2013; Wang et al., 2016) and flesh out in detail the taxonomy of CSR types and their respective predictors. This configuration-based typological approach helps to illustrate the intricate nature of firm’s CSR engagement. Third, we also contribute to the growing literature on the stakeholder-based view of CSR. Our study places personalized and SCL in the context of stakeholder theory (Freeman, 1984; Freeman et al., 2008; Samimi et al., 2022; Zhao et al., 2022) and shows that SCL is likely to foster CSR practices that focus on multiple stakeholders; other charismatic leaders are likely to exhibit personalized leadership by engaging in select CSR initiatives to target particular stakeholder groups who are beneficial for the leaders’ self-interests.

The pathway conceptualization has managerial implications as well. The focus on leader’s power motives and decision frames as drivers for CSR strategy would remind practitioners that CSR strategic transformation starts from the fundamental thinking about the objectives and purpose of the company. The recent writing on conscious capitalism and urgency of sustainability is a welcoming voice that challenges the conventional ideology of corporate objectives and encourages organizations to keep up with the time and critical issues. The configuration perspective of CSR strategic orientations can be a useful framework to holistically consider the various domains that constitute organization’s social performance.

Moving on to the limitations of this study, we proposed that, driven by their values and motives, SCL, NCL, and PCL are likely to engage in various types of CSR initiatives. However, one challenge that corporate leaders are constantly confronted with is resource constraint. Despite the will to do well and do good for all, oftentimes, the resource base of the firm is not expansive enough to facilitate every CSR activity. We did not explore how leaders deal with the trade-offs and prioritize their strategic choices among a range of CSR they wish to engage in. This limitation certainly prevented us from predicting the specificities of a firm’s CSR strategic balance (i.e., the amount of resources allocated to specific types of CSR). One possible way to deal with this issue in the future is to consider various contextual factors. Potential macro-level moderators (e.g., industry characteristics) and micro-level moderators (e.g., CEO characteristics) can facilitate understanding of more specific configurations of CSR activities for firms led by SCL, NCL, and PCL. Our model can also be expanded to include firm performance related to the SCL-CSR and PCL-CSR pathways in terms of both strategic and social outcomes.

Building on the idea of advancing the knowledge of CSR strategic configuration mentioned above, we believe that there are opportunities for making a theoretical contribution in the context of the stakeholder domain. CSR research has often been criticized for the lack of solid and coherent theoretical foundations (Jones et al., 2018; Hilliard, 2019). Though stakeholder theory continues to be the dominant paradigm in the field, the theory does not offer adequate explanations pertaining to the complex conflicts and interconnectedness among the stakeholder groups (Wang et al., 2020). Our study can be a starting point to examine the leaders’ response to the CSR pressures exerted by different stakeholder groups. For example, it might be interesting to explore how primary stakeholders react when firms led by socialized charismatic leaders focus on addressing the needs of secondary stakeholders and promote institutional CSR initiatives. Investigating such issues would not only bring to light the complex interactions between firms and stakeholders but also bolster the theoretical foundations of CSR research.

Further, for the purpose of gaining a fuller understanding of SCL, it might also be worthwhile to explore the theoretical overlap and divergence between SCL and another closely associated leadership style, responsible leadership. With a focus on social-relational and ethical obligations, responsible leadership has achieved prominence within the CSR domain (Maak and Pless, 2006; Miska and Mendenhall, 2018). Future research can bring to light the construct clarity of these two leadership styles, bridge these two leadership theories, and develop a thorough understanding of leader’s roles in CSR engagement.

## Author contributions

All authors listed have made a substantial, direct, and intellectual contribution to the work, and approved it for publication.

## Conflict of interest

The authors declare that the research was conducted in the absence of any commercial or financial relationships that could be construed as a potential conflict of interest.

## Publisher’s note

All claims expressed in this article are solely those of the authors and do not necessarily represent those of their affiliated organizations, or those of the publisher, the editors and the reviewers. Any product that may be evaluated in this article, or claim that may be made by its manufacturer, is not guaranteed or endorsed by the publisher.
